# Effect of Oral Bisphosphonates on Vertebral Fractures in Males Living with HIV: A Seven Year Study

**DOI:** 10.3390/jcm13216526

**Published:** 2024-10-30

**Authors:** Letizia Chiara Pezzaioli, Teresa Porcelli, Andrea Delbarba, Giorgio Tiecco, Francesco Castelli, Carlo Cappelli, Alberto Ferlin, Eugenia Quiros-Roldan

**Affiliations:** 1 Unit of Endocrinology and Metabolism, Department of Clinical and Experimental Sciences, University of Brescia, 25123 Brescia, Italy; letiziachiarapezzaioli@gmail.com (L.C.P.); carlo.cappelli@unibs.it (C.C.); 2 Unit of Endocrinology, Montichiari Hospital, ASST Spedali Civili Brescia, 25018 Montichiari, Italy; teresaporcelli@libero.it; 3 Unit of Endocrinology and Metabolism, Department of Medicine, ASST Spedali Civili, 25123 Brescia, Italy; delb.andrea@gmail.com; 4 Department of Clinical and Experimental Sciences, Unit of Infectious and Tropical Diseases, University of Brescia and ASST Spedali Civili, 25123 Brescia, Italy; g.tiecco@unibs.it (G.T.); francesco.castelli@unibs.it (F.C.); 5 Unit of Andrology and Reproductive Medicine, Department of Medicine, University of Padova, 35128 Padova, Italy

**Keywords:** oral bisphosphonates, bisphosphonates, HIV, PLWH, fractures, vertebral fractures

## Abstract

**Background:** Osteoporosis and vertebral fractures (VFs) are frequently observed in males living with HIV (MLWH). While bisphosphonates seem effective on bone mineral density (BMD) in MLWH, data on VFs are lacking. In this real-life longitudinal study performed on 118 MLWH (median age 53) who were followed-up for a median of 7 years, we aimed to evaluate the long-term efficacy of oral bisphosphonates on VFs in MLWH. **Methods:** The inclusion criteria were age >18, stable HIV infection, bisphosphonate-naïve, blood samples from the same laboratory, and three densitometries and morphometries performed with the same densitometer. **Results:** At baseline, VFs were detected in 29/118 patients (24.6%). Patients with VFs were older (*p.* 0.042), had longer HIV infection duration (*p.* 0.046) and antiretroviral exposure (*p.* 0.025), and demonstrated higher luteinizing hormone levels (LH, *p.* 0.044). Of the 29 patients with VFs at inclusion, 11 developed new VFs, of which 8 were under oral bisphosphonates (*p.* 0.018). Among the 89 without basal VFs, 11 developed VFs, of which 2 were under oral bisphosphonates. Patients with a worsened bone condition (regarding BMD and/or new VFs, n. 32) had more frequently high LH levels (>9.4 mIU/mL, *p.* 0.046) and higher HCV co-infection compared to patients with a stable bone condition (*p.* 0.045). It should be noted that 38.6% of patients discontinued oral bisphosphonates due to medical indication or personal choice, and 14.0% never started them. **Conclusions:** In conclusion, we found that oral bisphosphonates were not completely effective in preventing VFs, especially in patients with VFs at baseline; this is probably due to the multifactorial pathogenesis of fragility fractures in this population. A poor adherence to treatment and attention to gonadal function are also important issues in this population.

## 1. Introduction

The introduction of effective antiretroviral therapy (ART) has turned HIV into a chronic disease, associated with various complications and comorbidities, including osteoporosis [1]; this is a silent disease characterized by the deterioration of bone microarchitecture, low bone mineral density (BMD), and an increased risk of fractures [2].

People living with HIV (PLWH) have a lower spine and hip BMD, with a three-fold greater risk of osteoporosis with respect to controls [3,4]. Although data on vertebral fractures (VFs) are generally less conclusive, previous meta-analyses showed a two-fold increased risk of VFs compared to that of the general population [5,6,7]. Importantly, whether or not a low BMD leads to more fragile fractures in PLWH is still debated [5]. In fact, a low BMD explains only a 15% increase in fragile fractures, and the Fracture Risk Assessment Tool (FRAX) underestimates the risk of fractures in PLWH [8]. Indeed, the high frequency of fracture and low BMD among PLWH have a known multifactorial pathogenesis, with the involvement of both infection-related risk factors (HIV infection per se, ART exposure, chronic low grade inflammation, HBV and HCV co-infection, and low CD4 count) and traditional risk factors, such as low BMI, malnutrition, vitamin D deficiency, alcohol abuse, smoking, and hypogonadism [1,9,10].

Bisphosphonates are the first-line anti-osteoporotic treatment for osteoporosis [11]. Their action consists of inhibiting osteoclast activation, which helps to prevent bone resorption, with an improvement of BMD and a subsequent reduction in fracture risk [12]. The effect of bisphosphonates and the adherence to treatment can be indirectly measured by bone turnover markers; in fact, oral bisphosphonates determine a rapid decrease in both bone resorption and formation markers, and it has also been suggested that they can serve as a surrogate marker of fracture risk independently from BMD [13].

Treatment with bisphosphonates is known to have beneficial effects on BMD in PLWH, especially on the lumbar spine. A recent meta-analysis [14] found that the use of alendronate or zoledronate combined with calcium and vitamin D supplementation led to significant BMD improvement in PLWH on ART. Risedronate efficacy was investigated in one pilot study on males living with HIV (MLWH) [15], which showed an improvement in lumbar BMD according to gonadal status.

Denosumab was only recently evaluated as a therapeutic option in MLWH and proved to be as effective as zoledronate in maintaining/improving BMD [16].

For the general population, bisphosphonates have proven to be effective for fracture risk reduction [17]; however, data on the effect of bisphosphonates on fractures in the HIV population are lacking. Many variables are involved in the risk of fractures in PLWH, and establishing a clear correlation between therapy-related improvements in BMD and a reduced number of VFs is hard. Furthermore, previous studies had short-term follow-up times, which do not allow for the clarification of the potential role of bisphosphonates in preventing frailty fractures [14].

Therefore, we aimed to evaluate, in a real-life study with long-term follow-up times (up to 7 years), the efficacy of oral bisphosphonates (along with calcium and vitamin D supplementation) on VFs in MLWH. Furthermore, we aimed to investigate factors associated with bone health over time and response to anti-osteoporotic therapy.

## 2. Methods

### 2.1. Study Population

We performed a longitudinal study over a period from 2008 to 2020 in MLWH, who were consecutively sent from HIV specialists to the outpatient clinic of endocrinology.

The inclusion criteria were age >18 years old, documented HIV infection, stable ART, no personal history of malabsorption, no previous/ongoing treatment with drugs affecting bone health or bisphosphonates, blood samples carried out at a central hospital laboratory, and at least three DXA scans and three quantitative morphometric assays performed over time with the same densitometer (Explorer Hologic Inc., QDR-4500 W Waltham, MA, USA).

### 2.2. Data Collection and Definitions

VFs were assessed through a Genant semi-quantitative morphometric assay performed on images obtained from DXA by the same operator, measuring anterior, middle, and posterior vertebral heights and height ratios for each vertebra from T5 to L4 [18].

DXA results were expressed as BMD (g/cm^2^) and were collected for both spine, total hip, and femoral neck. For patients older than 50 years, a T-score (standard deviations—SDs—above or below sex and ethnicity matched the population at the peak of bone mass) was calculated. For patients younger than 50 years, both T- and Z-scores were collected, whereby a Z-score SD above or below sex and ethnicity matched a population of the same age. However, considering the small sample of patients who remained under 50 years throughout the duration of the study (n. 6), and to allow for a comparison between the densitometric parameters of the different visits, we considered only T-scores for statistical analysis. A T-score less than or equal to −2.5 SD at the spine or hip was defined as osteoporosis, whereas T-score values between −1 and −2.5 SD were diagnostic for osteopenia [19].

All blood samples were obtained between 8.00 and 10.00 a.m., after a 12 h overnight fast, and were analyzed at a central hospital laboratory of the ASST Spedali Civili Brescia, Italy. Blood and urinary samples were collected for the following biochemical assays at baseline evaluation: HBV and HCV serostatus, CD4 count, total testosterone (TT), sex-hormone-binding globulin (SHBG), follicle-stimulating hormone (FSH), and luteinizing hormone (LH). Serum and urinary calcium and phosphate, PTH, 25-OH-vitamin D, bone-alkaline phosphatase (bone formation marker), C-terminal telopeptide (bone resorption marker), and osteocalcin levels were collected both at baseline evaluation and at follow-up visits.

Hypogonadism was defined based on the concurrent presence of suggestive signs/symptoms (reduced libido, morning erections, and/or erectile function) and biochemical findings of low TT (measured with chemiluminescence microparticle immunoassay—CMIA) and/or cFT, calculated with Vermeulen equation (http://www.issam.ch/freetesto.htm, accessed on 30 July 2024), that combine TT, SHBG (measured with chemiluminescence immunoassay—CLIA), and albumin. If present, hypogonadism was classified according to LH levels (CMIA) into primary (high LH) and secondary (low/normal LH). The cut-off value for the lower limit of TT and cFT was 3.46 ng/mL and ≤65 pg/mL, respectively [20,21]. A normal range for LH was established between 1.5 and 9.4 mIU/mL [20,21]. Elevated LH with normal TT and cFT levels identified compensated (subclinical) hypogonadism. More details on the hypogonadism assessment in this population are shown elsewhere [22].

Information on smoking and drinking habits was collected. Patients were defined as current smokers if actively smoking at least 1 cigarette/day, or past smokers if they had quit smoking for at least one year. We considered patients as usual drinkers if they drink alcohol at least once a day, and occasional drinkers if they drink once a week or less.

Oral bisphosphonate therapy is offered free of charge to PLWH according to Italian appropriate prescriptive criteria. Weekly oral alendronate or weekly/monthly oral risedronate were prescribed, according to patients’ tolerance/preference.

### 2.3. Ethics

Ethical approval for this study was obtained from the Local Ethical Committee (Comitato Etico di Brescia, NP 3898) and informed consent was obtained from all participants.

### 2.4. Statistical Analysis

Statistical Package for the Social Sciences software IBM SPSS Statistics, Version 25.0 (Armonk, NY, USA) was used for statistical analysis. Since the variables were not normally distributed (Kolmogorov–Smirnov test was used), a comparison between the medians of the quantitative variables was performed with the non-parametric Kruskal–Wallis *H* test (followed by a post hoc Bonferroni test when a significant difference was found) or Mann–Whitney U test, as appropriate. Comparison between categorial variables was performed with Pearson’s Chi Square. In order to assess the change in radiologic, pharmacological, and biochemical parameters over time, Friedman’s test or Cochran’s *Q* test were performed, as appropriate. Multivariate models were designed, using logistic regression analyses, to investigate the clinical, therapeutic, biochemical, and radiological factors associated with bone health variation over time. All multiple regression analyses were preceded by univariate analyses to identify candidate predictive variables. *p* values ≤ 0.05 were considered significant.

## 3. Results

### 3.1. Baseline Characteristics

From 194 PLWH sent by the Unit of Infectious Diseases of Brescia, Italy, to the outpatient clinic of endocrinology, 118 met the criteria for inclusion. The median age of the whole population at the time of inclusion was 53 years old (IQR 49–57.3). The baseline characteristics of the study participants, according to the presence/absence of VFs at inclusion, are shown in Table 1. Patients with VFs were older (median age 55 vs. 52 years, *p.* 0.042) and had a longer duration of HIV infection (19 years vs. 11 years *p.* 0.046) and ART exposure (15 years vs. 5 years *p.* 0.025). Regarding gonadal function, at baseline, 26/118 (22.0%) patients had hypogonadism, including both the overt (low TT or cFT, independently from gonadotropin levels, 13/26 = 50%) and compensated forms (high LH with normal TT and cFT levels, 13/26 = 50%). The prevalence and type of hypogonadism was not different between patients with and without VFs, but LH levels were significantly higher in patients with VFs (*p.* 0.044) (Table 1).

### 3.2. Longitudinal Data

The median time between first and second visit was 2 years (IQR 2–3). The median time between first and latest available visit was 7 years (IQR 5–8). During the study period, all PLWH maintained undetectable plasmatic HIV RNA (<20 cp/mL), with a statistically significant median CD4 T cell increase (*p* < 0.001). The median CD4 T cell count was 631 mm/3 (31.7%), 678 mm/3 (32.4%), and 774 mm/3 (33%) at baseline, second, and latest visit, respectively. At baseline, 76 patients (64.4%) were on therapy with tenofovir disoproxil fumarate (TDF), while only 20 patients (16.9%) were on this form of therapy at the second visit and none at the end of follow-up.

#### 3.2.1. Patients with Vertebral Fractures at Baseline

**Figure 1:**
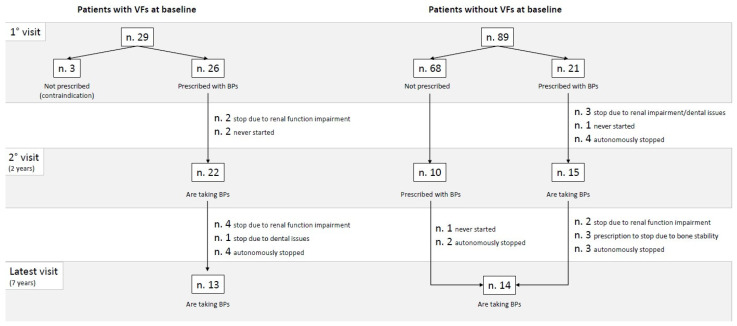
Flowchart of therapeutic management concerning bisphosphonates during follow-up, according to the baseline presence/absence of VFs at inclusion. Abbreviations: n.—number; VFs—vertebral fractures; BPs—bisphosphonates.

At inclusion, at least one VF was detected in 29/118 (24.6%) of the patients. Of them, 18/29 (62.1%) were osteoporotic, 11/29 (37.9%) had osteopenia, and none of them had normal BMD. Figure 1 shows the flowchart of therapeutic management concerning oral bisphosphonates during the follow-up of the patients with VFs at inclusion. Oral bisphosphonates were prescribed to 26 of the 29 patients with VFs, while the remaining 3 patients had pre-existing contraindications. By the time of the second visit (2 years), five patients had developed new VFs, of which two were regularly taking oral bisphosphonates, one had never received prescription due to contraindication, and two had to prematurely stop therapy due to renal function impairment. Moreover, two patients who received a prescription after their first visit never started therapy; therefore, at the second visit, 22 patients were still under treatment. Between the second and latest visit, five patients had to stop therapy due to nephrological/dental issues, and four patients autonomously discontinued taking oral bisphosphonates—all of them were exposed to continuative antiresorptive treatment for at least two years. By the time of the latest visit (7 years), 13 patients were still regularly taking therapy, and a further 6 patients had developed new VFs, all of them while on regular oral bisphosphonate treatment. Overall, during follow-up, 11 patients developed new VFs (11/29, 37.9%), of which 8 (72.7%) were under continuative antiresorptive treatment (*p.* 0.018), as reported in Table 2.

Table 3A shows data concerning calcium and cholecalciferol supplementation over time, as well as the variations in densitometric and biochemical parameters between the first, second, and latest visit.

After the first evaluation, up to 20% of the patients received calcium supplementation, and almost all the patients received cholecalciferol supplementation, without significant differences in terms of dosing over time.

During follow-up, BMD at both vertebral and femoral sites remained stable, even if the total hip T-score showed a progressive worsening from the first to the latest visit (*p.* 0.018). Considering the biochemical parameters, a progressive decrease in PTH (*p.* 0.010) and bone alkaline phosphatase (*p.* 0.031) was found, as expected with the introduction of the antiresorptive and suppletive therapeutic regimen [13].

#### 3.2.2. Patients Without Vertebral Fractures at Baseline

At inclusion, 89 patients did not have VFs. Of them, 26/89 (29.2%) were osteoporotic, 50/89 (56.2%) had osteopenia, and 13/89 (14.6%) had normal BMD. Figure 1 shows the oral bisphosphonate management concerning bisphosphonates during the follow-up of the patients without VFs at inclusion. Oral bisphosphonates were prescribed to 21 of the 89 patients with VFs, according to T-score and fracture risk assessment. By the time of the second visit (2 years), seven patients had developed new VFs, of which only one was regularly taking oral bisphosphonates. Furthermore, three patients had to prematurely stop therapy due to renal impairment/dental issues, four autonomously stopped oral bisphosphonates, and one never followed the prescription. Therefore, at the second visit, 15 patients out of the 21 prescribed were still under treatment. After the second visit, a further 10 patients were prescribed to start oral bisphosphonates, 6 due to the occurrence of VFs and 4 due to BMD worsening. Between the second and latest visit, three patients received a prescription to stop therapy due to the stability of BMD and the lack of VFs (“drug holiday”), two had to stop due to renal function impairment, five autonomously discontinued therapy, and one never started it. By the time of the latest visit (7 years), fourteen patients were still regularly taking therapy, and a further four patients had developed new VFs, of which one was regularly taking oral bisphosphonates, two had previously been treated but autonomously suspended it shortly after initiation, and one had never started it. Overall, during follow-up, 11 patients developed new VFs (11/89, 12.4%), of which only 2 (18.2%) were under continuative antiresorptive treatment (*p.* 0.811), as reported in Table 2.

Table 3B shows the variations in densitometric and biochemical parameters between the first, second, and latest visit, as well as data regarding calcium and cholecalciferol supplementation over time.

During follow-up, BMD at both vertebral and femoral sites remained stable, and spine T-score even improved (from −1.6 SD to −1.4 SD, *p.* < 0.01).

Considering biochemical parameters, a progressive decrease in PTH, bone alkaline phosphatase, and C-terminal telopeptide was observed (*p.* <0.01), as expected with the introduction of the antiresorptive therapeutic regimen [13]. Despite a slight lowering of 25-OH-vitamin D levels, almost all were supplemented with cholecalciferol during follow-up, with a significant and progressive dose increase over time (from 833 IU/day to 1190 IU/day, *p* < 0.01).

**Table 3:** Variation in densitometric and biochemical parameters between first, second, and latest visit available.

**(A) Patients with VFs at Baseline (n. 29)**				
**Patients with VFs at Baseline** **(n. 29)**	**At Inclusion**	**At Second Visit** **(2 years, IQR 2–3)**	**At Latest Visit** **(7 years, IQR 5–8)**	***p***
Supplementation				
Calcium, n (%)	3 (10.3)	4 (13.8)	6 (20.7)	0.311
Cholecalciferol, n (%)	17 (58.6)	28 (96.6)	25 (86.2)	**<0.01**
Cholecalciferol median daily dose (IU/day)	863 (800–1309)	893 (833–1190)	845 (743–1340)	0.629
Densitometric parameters				
Spine BMD	0.9 (0.8–1.0)	0.9 (0.8–1.0)	0.9 (0.8–1.0)	0.310
Spine T-score	−2.7 (−3.3; −1.5)	−2.1 (−3.0; −1.5)	−2.2 (−3.1; −0.8)	0.081
Total hip BMD	0.9 (0.7–0.9)	0.9 (0.7–0.9)	0.8 (0.7;1.0)	0.105
Total hip T-score	−1.5 (−2.6; −0.9)	−1.4 (−2.4; −0.6)	−1.6 (−2.0; −0.4)	**0.018**
Femoral neck BMD	0.8 (0.7–0.9)	0.8 (0.7–0.9)	0.7 (0.6–0.8)	0.094
Femoral neck T-score	−1.9 (−2.7; −1.4)	−1.9 (−2.6; −1.3)	−1.9 (−2.6; −1.3)	0.183
Biochemical parameters				
Calcium, serum (mg/dL)	9.3 (8.9–9.5)	9.2 (8.8–9.6)	9.5 (9.3–9.7)	0.434
Calcium, 24 h urine (mg/24 h)	243.5 (171.0–380.3)	235.0 (124.2–321.8)	220.0 (180.5–320.0)	0.345
Phosphate, serum (mg/dL)	2.7 (2.4–3.1)	2.4 (2.1–2.7)	2.5 (2.2–3.0)	0.056
PTH (pg/mL)	54.9 (43.5–66.6)	58.2 (42.5–66.7)	39.0 (30.3–51.0)	**0.010**
25-OH-vitamin D (ng/mL)	38.0 (27.2–50.0)	40.5 (28.3–49.0)	31.5 (24.0–42.0)	0.233
Bone alkaline phosphatase (IU/L)	35.5 (19.4–58.5)	19.5 (15.6–35.0)	11.1 (8.7–26.0)	**0.031**
C-terminal telopeptide (ng/mL)	0.3 (0.2–0.6)	0.1 (0.1–0.2)	0.2 (0.1–0.3)	0.206
**(B) Patients without VFs at baseline (n. 89)**				
**Patients Without VFs at baseline** **(n. 89)**	**At Inclusion**	**At Second Visit** **(2 years, IQR 2–3)**	**At Latest Visit** **(7 years, IQR 5–8)**	***p***
Supplementation				
Calcium, n (%)	2 (2.2)	14 (15.7)	17 (19.1)	**<0.01**
Cholecalciferol, n (%)	50 (56.2)	80 (89.9)	81 (91.0)	**<0.01**
Cholecalciferol median daily dose (IU/day)	833 (833–1071)	893 (833–1231)	1190 (833–1666)	**<0.01**
Densitometric parameters				
Spine BMD	1.0 (0.9–1.1)	1.0 (0.9–1.1)	1.0 (0.9–1.1)	0.177
Spine T-score	−1.6 (−2.5; −0.7)	−1.5 (−2.2; −0.7)	−1.4 (−2.2; −0.5)	**<0.01**
Total hip BMD	0.9 (0.8–1.1)	0.9 (0.8–1.0)	0.9 (0.8–1.0)	0.461
Total hip T-score	−0.9 (−1.6; −0.1)	−0.9 (−1.5; −0.2)	−1.0 (−1.5; −0.1)	0.371
Femoral neck BMD	0.8 (0.7–0.9)	0.8 (0.7–0.9)	0.8 (0.7–0.9)	0.771
Femoral neck T-score	−1.3 (−1.9; −0.8)	−1.3 (−1.7; 0.7)	−1.4 (−1.9; 0.8)	0.396
Biochemical parameters				
Calcium, serum (mg/dL)	9.3 (9.1–9.7)	9.3 (9.2–9.7)	9.3 (9.0–9.7)	0.862
Calcium, 24 h urine (mg/24 h)	203.0 (173.0–266.5)	269.0 (161.5–364.8)	211.0 (170.1–316.2)	0.069
Phosphate, serum (mg/dL)	2.4 (2.2–2.7)	2.4 (2.1–2.7)	2.5 (2.1–2.8)	0.189
PTH (pg/mL)	53.0 (36.1–71.5)	40.0 (31.2–58.0)	39.0 (30.1–56.5)	0.068
25-OH-vitamin D (ng/mL)	29.5 (17.3–37.0)	35.5 (27.0–44.0)	31.5 (26.0–39.9)	**<0.01**
Bone alkaline phosphatase (IU/L)	32.5 (25.3–46.5)	32.0 (26.3–43.0)	24.5 (14.6–35.0)	**<0.01**
C-terminal telopeptide (ng/mL)	0.5 (0.2–0.6)	0.3 (0.2–0.7)	0.3 (0.2–0.4)	**<0.01**

Variables are expressed as median (IQR), whereas categorial variables are expressed as absolute number (%), as appropriate. Comparisons are performed with Friedman’s test or Cochran’s *Q* test, as appropriate. A *p*-value ≤ 0.05 is considered significant in bold. Abbreviations: VF—vertebral fracture; BMD—bone mineral density; PTH—parathyroid hormone.

#### 3.2.3. Factors Associated with Bone Disease Evolution

Overall, during long-term follow-up, 22 out of 118 patients (18.6%) developed new VFs. Of them, 12 had osteopenia at baseline, of which 10 maintained stable BMD over time, and 2 even improved, whereas 10 were osteoporotic at baseline and maintained stable BMD over time. Compared to those who did not experience incident VFs, they more frequently reported the presence of VFs at baseline (50% vs. 18.8%, *p.* 0.002), and this association was also maintained in a subsequent multivariate logistic regression analysis (OR 4.12, CI 1.35–12.62, *p.* 0.013).

In Table 4A, we compared patients whose bone health worsened (in terms of both BMD, T-scores, and VFs) vs. those who maintained stable (or improved) bone condition during long-term follow-up. In evaluating bone loss progression over time, we considered as “worsened” patients those who transitioned from normal bone mineral density to osteopenia, and from osteopenia to osteoporosis, and also patients who developed new VFs, independently from BMD and T-score variation. Patients with a worsened bone condition over time (n. 32) more frequently showed LH levels > 9.4 mIU/mL (25.0% vs. 10.5%, *p.* 0.046), were more HCV co-infected (54.8% vs. 34.1%, *p.* 0.045), and more frequently received antiresorptive drugs (*p.* 0.008). They also had a lower CD4+ nadir count, close to significance (94.5 vs. 194.5, *p.* 0.053), which was also confirmed in the subsequent multivariate model (Table 4B). No other factors were found to be associated with bone worsening over time (Table 4B).

## 4. Discussion

In this longitudinal real-life study, we found that oral bisphosphonates may not be effective in preventing the occurrence of new VFs in a cohort of MLWH, despite maintaining a stable BMD over time, especially in patients already suffering from pre-existing VFs. Significantly, we reported a high burden of bone disease in our population, with VFs in almost a quarter of cases, and osteopenia/osteoporosis in nearly 90% of cases.

Our data on the prevalence of VFs at baseline substantially mirror previous findings in the literature [4,15,23,24], with a reported cumulative prevalence of morphometric VFs of 20.2% (CI 15.7–25.6) [6]. Patients presenting with VFs were older and had a longer history of both HIV infection and ART treatment, and patients with lower BMD values had a lower BMI, smoked more frequently, were drug users, and were HCV-co-infected, as already outlined by other authors [25,26]. Moreover, even if total testosterone levels did not differ between patients with or without VFs, fractured men had higher LH levels, suggesting an impairment of gonadal function, albeit still compensated [27]. These findings corroborate the multifactorial pathogenesis of osteoporosis in PLWH, related to both traditional and infection-related risk factors. Interestingly, we found that hypogonadism represents an independent risk factor, thus supporting the association between testis function and osteoporosis in MLWH [11,22,28,29].

During follow-up, BMD at both vertebral and femoral sites remained stable/slightly improved. Despite this, 22 patients (18.6%) developed new VFs; half of them already suffered from at least one VF at inclusion. In these patients, new VFs occurred regardless of bisphosphonate treatment, whereas, among the patients without VFs at inclusion who developed them during follow-up, only two were under oral bisphosphonate treatment.

Importantly, 38.6% of our patients discontinued oral bisphosphonates, both due to medical indication and to personal choice, and a further 14.0% never started them. These findings showed that poor adherence to therapy in PLWH is a considerable issue, also due to the burden of comorbidities, each requiring a specific treatment [30]. In a recent international survey [31], about a quarter of PLWH reported suboptimal adherence to antiretroviral therapy, mainly related to feelings of being overwhelmed from daily dosing, and partly due to drug-related side effects [32]. Focusing on anti-osteoporotic treatment, medication adherence is a remarkable issue even in the general population, especially in men. In fact, non-adherence to bisphosphonates in men ranges from one-third to two-thirds, with subsequent increased fracture risk [26,33]. Therefore, the active involvement of PLWH in a specific bone healthcare programme could help to improve overall adherence to osteo-active therapies.

Concerning the efficacy of bisphosphonates in maintaining/improving BMD, a meta-analysis [14] showed that alendronate and zoledronate—with calcium and vitamin D—were effective in improving the BMD in PLWH under ART up to 0.227 g/cm^2^, which is in substantial agreement with our results. However, data were not strong enough to establish whether bisphosphonates could reduce fracture risk, mainly due to the short-term follow-up performed by various studies and also because many of them did not include VFs as a primary end-point. The only study evaluating the prolonged use of zoledronate in PLWH [34] showed that the effect of two annual doses lasted for five years [35], and up to eleven years after the second infusion [36]. However, this study was relatively small and was performed on young men with a T-score below –0.5 SD, thus not allowing for either a generalization of their results or any assumption on efficacy in preventing fractures. Conversely, data on the efficacy of bisphosphonates in reducing vertebral fractures in the general male population are strong for zoledronate [37], which has also been confirmed for alendronate and risedronate [11].

There are limited data supporting the use of osteo-active drugs other than alendronate and zoledronate in PLWH, although there are also no data to suggest that these treatments would not be effective in relation to HIV [38]. In fact, risedronate has been investigated in a pilot study on MLWH by Pepe et al. [15], who proved it to be effective in significantly improving lumbar BMD, with better results in eugonadal patients than in hypogonadal ones. During the study period (24 months), no fragile fractures were observed, probably due to the short-term follow-up time and the relatively small number of patients included; therefore, no conclusion on its efficacy in preventing fractures could be made.

Concerning Denosumab, a recent study by Makras et al. [16] compared its efficacy with respect to zoledronate in improving BMD in 23 MLWH in an open label, 12-month cohort study. This study found that both Denosumab and zoledronate were effective in improving a BMD of over 5%, and no fragile fractures were observed during the study period. However, the small number of patients included and the short-term follow-up time do not allow for conclusions to be drawn on Denosumab’s efficacy in preventing long-term VF occurrence.

Another recent study [39] retrospectively evaluated the adherence and effectiveness of Denosumab treatment in Japanese PLWH, finding adherence rates at 12 and 24 months of 89.7% and 60.7%, respectively. Again, the small number of patients included and the lack of VF evaluation do not allow for a generalization; however, also considering the aforementioned adherence issue in this population, Denosumab sounds like a suitable option for osteoporosis treatment in MLWH, without forgetting to pay attention to its potential side effects, such as osteonecrosis of the jaw and atypical femoral fractures [40].

With respect to calcium and vitamin D supplementation, their benefit for PLWH has been shown, even if their use is not homogeneous between studies [14,38], and there is still no consensus on dosing and on an ideal threshold of vitamin D levels for improving BMD and preventing fractures [14]. However, a recent study with long-term follow-up times (up to 4–6 years), performed by Faber et al. [41,42], showed that calcium and vitamin D supplementation alone, when needed, increased spine BMD by 2% after 15 months, being stable afterwards, whereas hip BMD had no benefit. In our study, almost half of the patients were already adequately supplemented at inclusion, and calcium/vitamin D therapy was optimized for almost all patients during follow-up, generally reaching sufficient 25-OH-vitamin D values [43], which potentially might have partially helped in maintaining/improving BMD over time even in patients not exposed to bisphosphonates.

## 5. Limitations

The major limitations of our study include the absence of a control group of non-HIV patients. Moreover, the small numbers of MLWH who developed new fractures negatively affect the statistical power of our analysis, perhaps undermining the reliability of the findings. Finally, as this is a cohort study reflecting real-world conditions, our findings should be interpreted with caution due to the lack of randomization, which may introduce confounding-by-indication bias. Notwithstanding that, the strengths of our study include the long-term follow-up in a real-life setting, with a detailed evaluation of bone mineral status related to osteo-active treatments and instrumental and laboratory analyses performed at the same centre. Furthermore, we focused on VFs as a primary end-point, reporting novel and interesting findings in this field, highlighting the role of testis function and the issue of poor compliance to oral bisphosphonates in this population, which leads to early withdrawal and therefore potentially limit pharmacological efficacy.

## 6. Conclusions

In conclusion, in this longitudinal real-life study, we found that oral bisphosphonates, taken with adequate calcium and vitamin D supplementation, were able to maintain/improve BMD over time and to keep an overall sufficient 25-OH-vitamin D level, despite being less effective in preventing VFs, especially in patients already suffering from pre-existing VFs, probably due both to the multifactorial pathogenesis of fragile fractures in this population and to the difficult management of these patients (especially in terms of compliance). We also highlight the fact that gonadal function (expressed as LH levels) is an important issue in this population.

Therefore, our findings underscore the need for active screening and patient education, even when an anti-osteoporotic treatment is already ongoing, since it may be not enough to completely reduce the risk of fractures, which are associated with greater mortality rates.

## Figures and Tables

**Table 1:** Baseline characteristics of the study population. Comparison between patients with vertebral fractures with respect to patients without vertebral fractures at baseline.

**Patients** **(n. 118)**		**No Vertebral Fractures (n. 89)**	**Vertebral Fractures** **(n. 29)**	***p***
Age (years)		52.0 (48.5–57.0)	55.0 (50.5–62.0)	**0.042**
BMI (kg/m^2^)		24.9 (22.3–27.6)	24.0 (20.9–27.0)	0.183
>30, n (%)		10 (11.2)	1 (3.4)	0.210
Smoke	Current smoker, n (%)	29 (32.6)	11 (37.9)	0.632
	Past smoker, n (%)	5 (5.6)	2 (6.9)	
	Unknown, n (%)	21 (23.6)	8 (27.5)	
Alcohol	Usual drinker, n (%)	19 (21.3)	8 (27.5)	0.755
	Occasional drinker, n (%)	26 (29.2)	7 (24.1)	
	Unknown, n (%)	26 (29.2)	8 (27.5)	
Drug user, n (%)		26 (32.5)	12 (41.4)	0.116
HIV infection duration (years)		11.0 (7.0–20.0)	19.0 (10.5–25.0)	**0.046**
ART duration (years)		9.0 (5.0–16.0)	15.0 (8.0–19.0)	**0.025**
TDF exposure, n (%)		80 (89.9)	26 (89.7)	0.971
TDF therapy duration (years)		6.0 (4.0–8.0)	5.0 (2.0–8.0)	0.336
CD4 T cells nadir (cell/mm^3^)		190.5 (52.8–299.3)	113.5 (46.3–218.5)	0.260
CD4 T cells nadir (%)		15.1 (7.6–22.2)	14.0 (7.7–22.1)	0.812
AIDS, n (%)		27 (30.3)	10 (34.5)	0.479
TT (ng/mL)		6.6 (5.4–8.5)	6.9 (5.3–9.6)	0.268
SHBG (nmol/l)		56.0 (41.6–73.0)	74.5 (40–115.3)	0.131
cFT (pg/mL)		104.0 (77.5–120.0)	92.8 (75.3–128.0)	0.910
LH (mUI/mL)		4.8 (3.0–7.7)	7.4 (4.0–13.5)	**0.044**
FSH (mUI/mL)		5.8 (3.9–9.5)	6.9 (4.7–11.8)	0.347
Hypogonadism, n (%)		18 (20.2)	8 (27.6)	0.284
Hypogonadism class	Primary, n (%)	3 (16.7)	1 (12.5)	0.198
	Secondary, n (%)	8 (44.4)	1 (12.5)	
	Compensated, n (%)	7 (38.9)	6 (75)	
Chronic kidney disease, n (%)		7 (7.9)	4 (13.8)	0.281
Spine BMD (g/cm^2^)		0.97 (0.88–1.09)	0.86 (0.78–1.02)	**0.013**
Spine T-score (SD)		−1.6 (−2.5; −0.9)	−2.7 (−3.3; −1.5)	**0.005**
Total hip BMD (g/cm^2^)		0.92 (0.82–1.06)	0.83 (0.73–0.92)	**0.010**
Total hip T-score (SD)		−1.0 (−1.7; −0.1)	−1.6 (−2.5; −1.2)	**0.002**
Femoral neck BMD (g/cm^2^)		0.82 (0.71–0.93)	0.74 (0.67–0.81)	**0.040**
Femoral neck T-score (SD)		−1.3 (−1.9; −0.8)	−1.9 (−2.7; −1.4)	**0.001**
Bone status according to BMD	Normal BMD, n (%)	13 (14.6)	0 (0)	**0.003**
	Osteopenia, n (%)	50 (56.2)	11 (24.6)	
	Osteoporosis, n (%)	26 (29.2)	18 (62.1)	

Continuous variables are expressed as median (IQR), whereas categorial variables are expressed as absolute number (%), as appropriate. Comparisons for continuous variables were performed with Mann–Whitney U test. Comparison for categorial variables was performed with Pearson’s Chi Square. A *p*-value ≤ 0.05 is considered significant in bold. Abbreviations: BMI—body mass index; HIV—human immunodeficiency virus; TDF—tenofovir disoproxil fumarate; AIDS—acquired immunodeficiency syndrome; TT—total testosterone; SHBG—sex-hormone-binding protein; cFT—calculated free testosterone; LH—luteinizing hormone; FSH—follicle-stimulating hormone; BMD—bone mineral density; SD—standard deviation.

**Table 2:** Occurrence of VFs in patients with/without baseline VFs according to treatment with bisphosphonates.

VFs at Inclusion	Incident VFs During Follow-Up			*p*
	Total	Treatment with Bisphosphonates	No Treatment with Bisphosphonates	
Yes (n: 29)	11 (37.9%)	8 (72.7%)	3 (17.3%)	0.018
No (n: 89)	11 (12.4%)	2 (18.2%)	9 (81.8)	0.811

Abbreviations: VFs—vertebral fractures.

**Table 4:** (**A**) Comparison between patients whose bone health worsened, both in terms of BMD and vertebral fracture incidence, and those who maintained stable (or improved) bone condition during long-term follow-up. (**B**) Results of multivariate analysis, assessing factors associated with worsening of bone quality (BMD and vertebral fracture incidence).

**(A)**		**Worsened Bone Quality** **(BMD + VF)** **(n. 32)**	**Stable or Improved Bone Quality (BMD + VF)** **(n. 86)**	**Sig.**
Follow-up duration (years)		6 (5–8)	7 (5–8)	0.219
Age (years), median (IQR)		54 (48.5–61.5)	53 (49–57)	0.649
BMI (kg/m^2^), median (IQR)		24.9 (21.5–27.4)	24.6 (21.5–27.2)	0.848
HCV co-infection, n (%)		17/31 (54.8)	28/82 (34.1)	**0.045**
CD4 nadir count, median (IQR)		94.5 (39–242.5)	194.5 (56.5–305.8)	0.053
CD4 nadir (%), median (IQR)		11 (6.4–22.3)	15.6 (8.8–22.1)	0.109
TDF exposure, n (%)		28/31 (90.3)	73/81 (90.1)	0.975
Hypogonadism, n (%)		9/32 (28.1)	17/81 (21)	0.417
BMD at baseline	Normal, n (%)	5/32 (15.6)	8/86 (9.3)	0.616
	Osteopenia, n (%)	16/32 (50)	45/86 (52.3)	
	Osteoporosis, n (%)	11/32 (34.4)	33/86 (38.4)	
Prevalent vertebral fractures, n (%)		11/32 (34.4)	18/86 (20.9)	0.132
Bisphosphonate exposure, n (%)		19/32 (59.4)	28/86 (32.6)	**0.008**
High LH (>9.4 IU/L)		8/32 (25.0)	9/86 (10.5)	**0.046**
**(B)**		**Bone Quality Worsening During Follow-Up**		
		**OR (95% CI)**		**Sig.**
Follow-up duration		1.16 (0.89–1.52)		0.264
Age		1.01 (0.93–1.08)		0.867
BMI		1.00 (0.87–1.16)		0.963
CD4 nadir		0.99 (0.98–1.00)		0.055
CD4 nadir (%)		1.04 (0.96–1.12)		0.295
HCV co-infection		2.39 (0.81–7.03)		0.114
TDF exposure		0.57 (0.10–3.13)		0.517
Prevalent VFs		0.65 (0.17–2.40)		0.514
Bisphosphonate exposure		4.38 (1.34–14.30)		**0.014**
Hypogonadism		1.21 (0.40–3.67)		0.736
High LH (>9.4 IU/L)		2.17 (0.24–19.89)		0.493

Continuous variables are expressed as median (IQR), whereas categorial variables are expressed as absolute number (%), as appropriate. (**A**) Comparisons for continuous variables are performed with the Mann–Whitney U test, whereas comparisons for categorial variables are performed with Pearson’s Chi Square. A *p*-value ≤ 0.05 is considered significant in bold. (**B**) Multivariate logistic regression analysis. Abbreviations: BMI—body mass index; HCV—hepatitis C virus; BMD—bone mineral density; TDF—tenofovir disoproxil fumarate; VF—vertebral fracture; LH—luteinizing hormone.

## Data Availability

The data that support the findings of this study are available from the corresponding author upon reasonable request.
